# Supplementary material for: Cortical motor activity modulates respiration and reduces apnoea in neonates
Source: eLife. 2026 May 12;14:RP107081. doi: 10.7554/eLife.107081 (PMC13167109; doi:10.7554/eLife.107081)
Supplement: Peer review history

# Editor report: eLife Assessment

**DOI:** 10.7554/eLife.107081.3.sa0

**Authors:** Nicole C Swann

**Affiliations:** University of Oregon United States

**Keywords:** Convincing, Fundamental

Zandvoort and colleagues have used an innovative approach to study respiration-brain coupling in the context of apnoea in human newborns. This **fundamental** question is supported with **convincing** data and analyses. Having addressed all the reviewer comments, there was a general consensus that this work will be of great interest, not only to neonatal clinicians and physiologists, but also broadly to anyone interested in brain-body interactions.

# Referee report: Reviewer #1 (Public review)

**DOI:** 10.7554/eLife.107081.3.sa1

Summary:

The authors investigated the extent to which phase-amplitude coupling (PAC) of respiratory and electrophysiological brain activity recordings was related to episodes of life-threatening apnoea in human newborns.

Strengths:

I want to commend the authors for acquiring unique and illuminating data; the difficulty in recording and handling these data has to be appreciated. As far as I can tell, Zandvoort and colleagues are the first to provide robust evidence for respiration-brain coupling in newborns. Their creative use of the phase-slope index for peripheral-central interactions is innovative and credible. If proven to be robust, the authors' findings have important implications well beyond the field of brain-body research.

Comments on revisions:

I would like to thank the authors for a careful revision and additional clarifications; I have no further questions.

# Referee report: Reviewer #2 (Public review)

**DOI:** 10.7554/eLife.107081.3.sa2

Summary:

The author's central hypothesis was that the strength of cortico-respiratory coupling in infants is negatively associated with apnoea rate. To prove this, they first investigated the existence of cortico-respiratory coupling in premature and term-born infants, the spatial localisation of the cortical activity and its relationship with the phase of the respiratory cycle, and the directionality of coupling.

Strengths:

The researchers used synchronised EEG and impedance pneumography to detect the phase amplitude coupling.

They have studied a wide range of gestations, from 28 weeks to 42 weeks, including males and females. Their exclusion criteria ensured that healthy babies were studied and potential confounders of impaired respiratory activity were avoided. Their sequential approach in addressing the objectives was appropriate.

Weaknesses:

As a neonatal clinician and neuroscientist, I have commented based on my expertise. I have not commented on signal processing.

There are no major weaknesses to the study. Some minor weaknesses include:

(1) Data relating to the cortical oscillations and the respiratory phase is given. However, whether this would lead to their hypothesis that the strength of cortico-respiratory coupling is negatively associated with apnoea rate is unclear. What preceding data enabled the authors to link the strength of coupling to the rate of apnoea?

(2) If we did not know of data showing the existence of cortico-respiratory coupling in newborn infants, then should it not be the first research question to examine?

(3) What are the characteristics of the infants who contributed data to establish the cortico-respiratory coupling (Figures 2 and 3)?

(4) Although it is the most plausible direction of the relationship, with neural activation driving respiratory muscle contraction, how can the authors prove this with their data? Given that they show coherence between signals, how do we know that the cortical signal precedes the respiratory muscle contraction?

(5) Apgar score is an ordinal variable. The authors should summarise this as median (range).

Comments on revisions:

All the weaknesses are adequately addressed. No more comments

# Author comment: Author response

**DOI:** 10.7554/eLife.107081.3.sa3

**Authors:** Coen Zandvoort, Fatima Usman, Shellie Robinson, Odunayo Fatunla, Eleri Adams, Kyle T Pattinson, Simon F Farmer, Caroline Hartley

**Affiliations:** University of Oxford Oxford United Kingdom; University of Oxford Oxford United Kingdom; University of Oxford Oxford United Kingdom; University of Oxford Oxford United Kingdom; University of Oxford Oxford United Kingdom; University of Oxford Oxford United Kingdom; University College London London United Kingdom; University of Oxford Oxford United Kingdom

The following is the authors’ response to the original reviews

**Public Reviews:**

**Reviewer #1 (Public review):**

Summary:

The authors investigated the extent to which phase-amplitude coupling (PAC) of respiratory and electrophysiological brain activity recordings was related to episodes of life-threatening apnoea in human newborns.

Strengths:

I want to commend the authors for acquiring unique and illuminating data; the difficulty in recording and handling these data has to be appreciated. As far as I can tell, Zandvoort and colleagues are the first to provide robust evidence for respiration-brain coupling in newborns. Their creative use of the phase-slope index for peripheral-central interactions is innovative and credible. If proven to be robust, the authors' findings have important implications well beyond the field of brain-body research.

Weaknesses:

While the analyses were overall competently conducted and well-justified, I was not entirely convinced by a few methodological choices, specifically (i) the computation of PAC surrogates, (ii) details of the linear mixed-effects model, and (iii) the electrode selection for linking phase-amplitude coupling to apnoea frequency.

Thank you for your kind comments and helpful review of our paper. We have now clarified computation of PAC surrogates, added further details of the linear-mixed effects models and calculated the link between the strength of the cortico-respiratory coupling (phase-amplitude coupling) and apnoea rate with data acquired at all electrodes. We provide further details for each of these in response to your ‘Recommendations for the authors’.

**Reviewer #2 (Public review):**

Summary:

The author's central hypothesis was that the strength of cortico-respiratory coupling in infants is negatively associated with apnoea rate. To prove this, they first investigated the existence of cortico-respiratory coupling in premature and term-born infants, the spatial localisation of the cortical activity and its relationship with the phase of the respiratory cycle, and the directionality of coupling.

Strengths:

The researchers used synchronised EEG and impedance pneumography to detect the phase amplitude coupling.

They have studied a wide range of gestations, from 28 weeks to 42 weeks, including males and females. Their exclusion criteria ensured that healthy babies were studied and potential confounders of impaired respiratory activity were avoided. Their sequential approach in addressing the objectives was appropriate.

Weaknesses:

As a neonatal clinician and neuroscientist, I have commented based on my expertise. I have not commented on signal processing.

I did not identify any major weaknesses in the study. Some minor weaknesses include:

(1) Data relating to the cortical oscillations and the respiratory phase is given. However, whether this would lead to their hypothesis that the strength of cortico-respiratory coupling is negatively associated with apnoea rate is unclear. What preceding data enabled the authors to link the strength of coupling to the rate of apnoea?

(2) If we did not know of data showing the existence of cortico-respiratory coupling in newborn infants, then should it not be the first research question to examine?

(3) What are the characteristics of the infants who contributed data to establish the cortico-respiratory coupling (Figures 2 and 3)?

(4) Although it is the most plausible direction of the relationship, with neural activation driving respiratory muscle contraction, how can the authors prove this with their data? Given that they show coherence between signals, how do we know that the cortical signal precedes the respiratory muscle contraction?

(5) Apgar score is an ordinal variable. The authors should summarise this as median (range).

Thank you for your useful comments. We have revised the manuscript to address these comments and improve the clarity.

(1) We agree that proceeding data leading to the hypothesis that the strength of cortico-respiratory coupling is negatively associated with apnoea rate is limited. We have clarified in the introduction that adult studies have previously suggested that cortical motor activity may prevent hypoventilation and apnoea seen in patient groups. We have also added further clarification to our hypothesis. In the introduction we now state:

“In adults with congenital central hypoventilation syndrome or obstructive sleep apnoea, a respiratory-linked increase in cortical motor activity suggests that the motor cortex plays an important role in maintaining autonomous respiration, with the authors postulating that cortico-respiratory drive whilst participants are awake may prevent the hypoventilation/apnoea observed in these patients whilst they are asleep.”

And later:

“We hypothesised that cortico-respiratory coupling occurs in newborns and that the strength of cortico-respiratory coupling is negatively associated with apnoea rate (in line with the suggestions made from studies of adults with congenital central hypoventilation syndrome[6] and obstructive sleep apnoea[7]).”

(2) We agree that this was the first research question we examined. We have clarified this in the introduction, now re-writing the hypothesis and aims to state “We hypothesised that cortico-respiratory coupling occurs in newborns and that the strength of cortico-respiratory coupling is negatively associated with apnoea rate (…). To this end, we first examined whether cortico-respiratory coupling exists in both premature and term infants.”

(3) Figures 2 and 3 used the full dataset. We have clarified this in the Figure captions by stating: “For all panels, data included is from 68 infants (28-42 weeks postmenstrual age [PMA] at time of recording) on 104 recording occasions. See Table 1 for further clinical and demographic characteristics.”

(4) We used a cross-frequency version of the phase-slope index to quantify the directionality and strength of information flow between cortical and breathing time series (Figure 3C,D). The phase-slope index investigates phase lags and how these change over narrow frequency ranges by examining the slope of the phase spectrum of their complex coherency. This indicates whether one signal leads or trails another signal (and thus indicating directionality). However, we agree (and as was also noted by Reviewer 3) that this analysis does not ‘prove’ directionality as other factors may influence the analysis. We have added the following to the text to address this point:

“However, caution is needed in the interpretation of these results as signal processing techniques such as the phase-slope index estimate directionality but do not confirm causality. Rather, they show a statistical relationship which can be influenced by a multitude of factors (e.g., signal-to-noise ratio and preprocessing steps). Nevertheless, the results suggest that cortical activity may precede respiration in newborns. Future work is needed to confirm this association by, for example, employing other metrics to estimate directionality, such as the time-lagged cross-correlation and Granger causality and through direct mechanistic studies.”

(5) We have revised Table 1 so that Apgar scores are provided as median and interquartile range.

**Reviewer #3 (Public review):**

Summary:

This is a strong and important report that presents a framework for understanding cortical contributions to neonatal respiration. Overall, the authors successfully achieved their goal of linking cortical activity to respiratory drive. Despite the correlational nature of this study, it is a crucial step in establishing a foundation for future work to elucidate the interaction between cortical activity and breathing.

Strengths:

(1) The introduction and use of workflows that establish correlational relationships between breathing and brain activity.

(2) The execution of these workflows in human neonates.

Weaknesses:

Interpretations related to causal inference, confounds of sleep and caffeine, and the spatial interpretation of EEG data need to be addressed to ensure that the data appropriately support the conclusions.

Thank you for your useful comments. We have now substantially revised the manuscript in relation to causal inference and our interpretations of the data, in particular adding further detail to the discussion with regards to the limitations of our approach and revising wording that has causal implications. We provide more detail in response to your ‘Recommendations for the authors’.

**Recommendations for the authors:**

**Reviewer #1 (Recommendations for the authors):**

I want to elaborate on the three points of methodological criticism, and my apologies in case I have some misconceptions:

(1) It seems like the surrogate distribution to determine PAC significance was computed by shuffling EEG segments and recomputing PAC each time. Surrogate computations are a difficult topic when handling signals as regular as respiration time series. However, random shuffling of data segments is almost always an overly liberal approach (except for trial-based data) since it destroys the temporal autocorrelation of the underlying signal. As the resting-state data in the present study were per sé continuous (and just segmented for analytical purposes), I am not convinced that random shuffling provides an adequate control. Could the authors either (a) provide convincing evidence that the temporal autocorrelation of verum and surrogate time series did not differ from one another, or (b) conduct additional control analyses based on an alternative approach, e.g., by constructing surrogate respiration phase vectors and recomputing PAC accordingly? We have had good experiences with the IAAFT approach (outlined in Kluger et al., Nat Comms 2023), but others certainly exist.

Thank you for this important comment on the construction of surrogates. We agree that it is essential for any surrogate approach that it destroys the cross-signal coupling whilst preserving the signals’ internal structure (e.g., autocorrelation, spectral profile, and non-stationarities) as much as possible. We apologies for not describing this clearer in the initial manuscript, but we want to clarify that in the surrogate analysis, we did not shuffle time points/segments within EEG trials itself. Instead, we permuted the trial order so that respiration trial T1 was paired with an EEG trial other than T1. This leaves the 4-sec segments used in the PAC analysis unaltered. This surrogate technique preserves the important internal properties of each signal (within-trial autocorrelation, auto-spectra and power distribution of the signals) while destroying the cross-signal alignment across trials, and thus the trial-wise phase locking (e.g., coherence) between respiration and EEG. We have clarified this in the manuscript as follows:

“The surrogate analysis was performed by randomly permuting the trial (4-s segment) order of the EEG amplitude while leaving the respiration trial order unchanged (i.e., respiration segment S1 was paired with an EEG segment Sj, j ≠ 1). Importantly, no temporal samples were shuffled within segments. Thus, the full within-segment temporal structure, including autocorrelation and spectral profile (auto-spectra), was preserved for both signals. This permutation destroys trial-wise cross-signal phase alignment (and therefore coherence) while retaining the intrinsic dynamics of each signal.”

(2) The LMEM approach is very sound, but it seems like ID was the only random effect included in the model. Could the authors clarify whether multiple sessions from individual neonates were considered or whether each ID was only represented once? In case of the former, one possibility would be to include 'session' as an additional random effect; otherwise, the group statistic could be biased. Many thanks in advance for providing insight on this.

Thank you for this important point. Of the 68 infants included in the study, 49 only had a single session. The remaining 19 infants had between 2 – 5 sessions included. Given that most infants only had a single session it is not possible to identify random effects of session reliably and so we have not included session as a random factor. Moreover, postmenstrual age [PMA] (which is related to session order within a subject and is likely a more reliable indicator of variance given that sessions were not at fixed intervals) is already included as a factor in the analysis. Indeed, session ID is not a distinct source of clustering and will be indistinguishable from subject and PMA variance.

In relation to this question, we carefully checked the analysis and realised that we had included infant with a random effect of both slope and intercept. Given that most infants have only one session the random effect of slope cannot be estimated and so we have now removed this from the analysis leading to very minor changes in the results (and no changes in the interpretation). We have clarified in the manuscript that “Infant ID was included as a random effect acting on the intercept.”

(3) It is not entirely clear to me why the authors selected the two electrodes with the strongest overall PAC for the analysis of apnoea frequency. Why not consider all electrodes individually? What is the worry/hypothesis regarding electrodes with low PAC - would one not expect simply to find no relationship with apnoea frequency, and would that information not be instructive? Again, I want to thank the authors in advance for their take on this comment.

We initially included only the two electrodes with the strongest coupling as we would not expect a relationship with apnoea rate at those electrodes without significant coupling (as you say). For completeness, we have now included the relationships with all electrodes individually in Supplementary Figure S4. As expected, the relationship between apnoea rate and coupling (coherence) was not significant for the electrodes without strong coupling.

**Reviewer #3 (Recommendations for the authors):**

Major Comments:

(1) Causal Language and Overinterpretation are evident throughout the manuscript. The manuscript repeatedly uses language suggesting causality (e.g., "cortical motor activity reduces apnoea"), despite the correlational nature of the findings.

It is recommended that the authors reframe their claims in the abstract and discussion to clarify that the observed associations do not establish causal influence. For example, Abstract: "...revealing novel mechanistic insight....". This correlational observation does not reveal a mechanism but rather supports the concept of mechanistic interactions.

Thank you for this important point. We have now rephrased the manuscript throughout, particularly in the abstract and results/discussion. We have also added the following sentences to the discussion to address the point on causation:

“Nevertheless, it is important to recognise that a limitation of this analysis is that correlation does not imply causation, and future mechanistic studies are required to determine whether and how cortico-respiratory coupling plays a role in reducing apnoea in infants.”

And later:

“The limitations of our study need to be considered, and in particular, directionality of the cortico-respiratory coupling, improved spatial localisation, and a direct mechanistic link between cortico-respiratory coupling and apnoea rate, should be investigated in future work.”

(2) Potential Confounding by Sleep State and Caffeine. Sleep state is a significant determinant of apnoea occurrence and EEG frequency composition, yet no objective sleep-state classification is incorporated. Similarly, caffeine, administered in ~50% of recordings, is a potent respiratory stimulant. A reanalysis of the data, incorporating sleep proxies (e.g., EEG spectral ratios, delta/theta dominance) and caffeine exposure as covariates or stratification factors in the PAC-apnoea models, should be performed.

Sleep state: A limitation of our work is that we did not record sleep state and unfortunately, we do not have anyone trained to annotate sleep states from EEG recordings in our research group. We have added the following to the discussion to address this:

“It is known that most apnoeas in infants occur during active sleep[6][30] and delta- and theta-band frequencies in EEG are strongly related to sleep state[31]. A limitation of our study is that we did not record the sleep state of the infant.”

Caffeine: We agree that caffeine is a respiratory stimulant and, hence, it is important to consider this effect. Moreover, those infants prescribed caffeine are those who are at greatest risk of apnoea and so it is of interest to determine whether the relationship between PAC and apnoea rate occurs in those infants receiving caffeine treatment. We conducted a stratified analysis to address this point, now providing an additional Supplementary Figure.

(3) Directionality Inference from Phase-Slope Index. While PSI suggests a lead-lag relationship, it does not confirm causality and may be influenced by signal-to-noise or preprocessing steps. Validation PSI findings using additional metrics (e.g., time-lagged cross-correlation or Granger causality) or, at a minimum, temper interpretations of cortical "driving" respiration.

We agree that the PSI (and other metrics such as Granger causality) may be influenced by a range of factors. We have therefore changed the wording throughout and also added the following:

“However, caution is needed in the interpretation of these results as signal processing techniques such as the phase-slope index estimate directionality but do not confirm causality. Rather, they show a statistical relationship which can be influenced by a multitude of factors (e.g., signal-to-noise ratio and preprocessing steps). Nevertheless, the results suggest that cortical activity may precede respiration in newborns. Future work is needed to confirm this association by, for example, employing other metrics to estimate directionality, such as the time-lagged cross-correlation and Granger causality and through direct mechanistic studies.”

(4) Limited EEG Spatial Resolution. The attribution of CRC to "cortical motor areas" is overstated, given the use of only 8 EEG electrodes, which provides limited spatial coverage. Avoid overly precise interpretations regarding cortical localization unless source localization or higher-density EEG data are available.

We have added the following to specifically address this limitation.

“It is important to note that the number of electrodes in our montage is limited (with only 8 recording electrodes), and so source localisation was not possible; higher-density recordings are warranted to confirm whether the motor cortex plays a role.”

We have also changed the wording in the summary paragraph and abstract to add this limitation and reworded throughout the manuscript to highlight the limitations of our study.

Minor Comments

(1) Consider color-coding individual points in Figure 4A by PMA or caffeine status to visually disambiguate potential age-related or pharmacological effects.

We agree that this provides additional visual information and have colour-coded the points in Supplementary Figure S6 according to caffeine status.

(2) Clearly define PAC versus CRC. These are used interchangeably. Readers may benefit from a more consistent and precise usage, especially in the abstract.

Thank you for noticing this. We have revised the terms where necessary throughout, and the abstract and introduction to read:

“Using simultaneous electroencephalography (EEG) and impedance pneumography we investigated interactions between cortical and respiratory activity (known as cortico-respiratory coupling) using phase-amplitude coupling.”

“Recently, it was proposed that communication between the cortex and lungs, known as cortico-respiratory coupling, can be identified and quantified through phase-amplitude coupling. This functional coupling arises when the amplitude of cortical activity is modulated by the respiration phase, or vice versa. Phase-amplitude coupling is typically quantified using non-invasive recordings capturing respiratory and neural activity (e.g., magneto- or electroencephalography [EEG]).”

(3) Clarify the overlap with previously published datasets (line 358). Are any EEG-apnoea associations re-analyses of data published in Zandvoort et al., 2024?

We have amended this sentence to explain that the previous study did not investigate respiration/apnoea. We now state:

“Parts of this dataset have been reported earlier in Zandvoort et al. [33] to address a different research question (this study investigated the development of sensory-evoked potentials, which were also recorded in these infants; it did not explore respiration).”
